# Deliberate Soccer Practice Modulates Attentional Functioning in Children

**DOI:** 10.3389/fpsyg.2020.00761

**Published:** 2020-05-12

**Authors:** Consuelo Moratal, Juan Lupiáñez, Rafael Ballester, Florentino Huertas

**Affiliations:** ^1^Department of Physical Education and Sport Sciences, Catholic Universiy of Valencia San Vicente Mártir, Valencia, Spain; ^2^Department of Experimental Psychology, Mind, Brain and Behavior Research Center (CIMCYC), University of Granada, Granada, Spain

**Keywords:** executive control, orienting, alerting, childhood, team sport

## Abstract

The main purpose of this study was to explore the association between the regular practice of open-skill sports (i.e., soccer) and executive control, along with other attentional functions (i.e., alerting and orienting) during preadolescence. The study was conducted on 131 participants (70 non-athletes and 61 soccer players). To measure cognitive performance, participants performed the Attentional Network Test—Interactions (ANT-I) task. Compared to non-athletes, soccer players showed overall faster responses and better executive control (e.g., reduced interference from distractors). Overall, our results provide new empirical evidence supporting the positive association between regular sports practice and cognitive performance, and more specifically executive functions. However, is important to note that the relationship between regular sport practice and cognition is complex and multifactorial. Our findings can be partly explained by the “cardiovascular fitness hypothesis” and the “cognitive component skills approach,” suggesting that an externally paced sport environment with high physical fitness and perceptual–cognitive demands may be an appropriate setting to optimize the development of cognitive functioning during early adolescence.

## Introduction

The physical, psychological, and socio-affective benefits of physical activity have been widely described in numerous scientific studies (see reviews by Eime et al., 2013; Warburton and Bredin, 2017; Milanović et al., 2019). Regarding benefits at the cognitive level, many studies have explored this association in older adults (see the review by Northey et al., 2018). In addition, a great deal of research has recently been conducted on children and young adults, revealing the positive influence of regular physical exercise on cognitive performance (see reviews by Donnelly et al., 2016; Bidzan-Bluma and Lipowska, 2018; de Greeff et al., 2018). However, not all physical activities (i.e., working, free play, and deliberate practice) are the same. Ericsson et al. (1993) defined the term “deliberate practice” in terms of the quality of the practice time, only considering as deliberate practice highly structured activities that have been specially designed to develop specific goals and, consequently, to improve the current level of performance. In our investigation, we will focus our interest on the study of the association between deliberate soccer practice and attentional functioning in children.

Among the various cognitive functions that have been studied, researchers have particularly focused on understanding the relationship between regular exercise and executive control, especially in older adults (see the review by Sanders et al., 2019) and other age groups (see reviews by Best, 2010, with children, and Guiney and Machado, 2013, with other age groups).

Executive control is defined as a set of high-level cognitive functions involved in the planning, initiation, sequencing, and analysis of complex, goal-driven behaviors (Diamond, 2013). These cognitive functions are grouped into three types of sub-processes: (a) inhibitory control, which involves the ability to filter out irrelevant information to avoid unnecessary responses and selectively maintain attention and control over one’s actions; (b) working memory, which reflects the ability to keep in mind and manage information while performing complex cognitive tasks; and (c) cognitive flexibility, that is, the ability to restructure information and knowledge to efficiently adapt to various situational demands. In the sports context, executive control allows athletes to make quick decisions when faced with high cognitive demands. It also enables them to optimize their performance and is essential for their success, especially in externally paced sports with rich and complex stimuli (Vestberg et al., 2017).

The relationship between regular physical activity and executive control has been studied from different methodological approaches. The dominant approach has been to analyze cognitive performance using tasks that measure executive functions in laboratory contexts (e.g., Belsky et al., 2015; Chekroud et al., 2018). However, few studies have explored the association between regular practice of a specific sport and executive control, while simultaneously taking into account other attentional functions such as those described by Posner (1980): alerting, orienting, and executive control. Sports in general, and more specifically externally paced ones, are a rich environment to test this global function of attention given the complexity of the present stimuli and the decision-making processes involved. Therefore, our research focused precisely on analyzing the association between regular practice of a predominantly externally paced or open-skill sport such as soccer and executive control in the context of other attentional networks.

Regarding the positive link between regular physical exercise and executive control in children (see reviews by Guiney and Machado, 2013; de Greeff et al., 2018), most studies have focused on exploring the mediating effect of cardiovascular fitness (Buck et al., 2008; Hillman et al., 2009; Chaddock et al., 2010, 2012; Castelli et al., 2011; Davis et al., 2011; Pontifex et al., 2011; Voss et al., 2011). Overall, a positive correlation has been observed between the level of cardiovascular capacity and the performance of executive functions. These findings are based on the “cardiovascular fitness hypothesis” (North et al., 1990; Voss et al., 2011), according to which improved aerobic abilities, which are inherent to regular physical exercise, are the physiological mediator that determines cognitive benefits. More specifically, Hillman et al. (2009) and Pontifex et al. (2011) used various types of flanker tasks. They observed that preadolescent children with lower aerobic fitness showed less accuracy than those with higher fitness levels, although their speed of response was not affected. By contrast, studies such as that of Kimball (2009) have found no difference in antisaccade or flanker tasks between participants with regular aerobic practice and control participants with no regular practice. On the other hand, many studies have analyzed the relationship between sport expertise and executive control in young people, showing benefits in participants with greater skills in sports such as fencing (Chan et al., 2011), basketball (Alarcón et al., 2017), or badminton (Yu et al., 2017). These findings have been explained on the basis of the “cognitive component skills approach,” according to which sports training acts as a means to improve brain plasticity and develop certain cognitive functions more efficiently (Voss et al., 2010; Scharfen and Memmert, 2019a). This would explain the “specialization” in attentional performance achieved by practicing predominantly open-skill sports. Focusing on studies carried out on children, Verburgh et al. (2014) observed that highly skilled soccer players showed greater motor inhibition than amateur soccer players but no differences in executive attention or visuospatial working memory. In a later study, these authors compared the sport practice level of three groups of children (i.e., highly skilled soccer players with very frequent practice, amateur players with regular practice, and non-athletes with no practice) on motor inhibition, short-term memory, working memory, and the three attentional networks: alerting, orienting, and executive attention (Verburgh et al., 2016). Their findings showed that the higher the levels of sports practice, the better the measured motor inhibition and short-term memory. Moreover, amateur soccer players outperformed non-athletes in both short-term memory and working memory. More recently, Scharfen and Memmert (2019b) reported a positive relationship between performance in basic cognitive skills (i.e., working memory, perceptual load, multiple object tracking, and attention window) and specific motor abilities (i.e., sprint, change of direction, dribbling, ball control, shooting, and juggling) in a small sample of elite youth soccer players (*n* = 15, *M*_age_ = 12.72).

Scarce evidence exist on the link between regular physical exercise and exogenous spatial orienting, yielding variable results (Nougier et al., 1996; McAuliffe, 2004; Verburgh et al., 2014). Nougier et al. (1996) found that young adults who practiced predominantly open-skill sports showed greater flexibility and attentional control than their counterparts who practiced predominantly closed-skills sports. In the same age group, using a spatial cueing task, McAuliffe (2004) found that cueing effects were greater in university volleyball players than in non-athletes. At younger ages, only the study by Verburgh et al. (2014), comparing a sample of highly skilled vs amateur soccer players (8–12 years old), reported no differences between groups in attentional orienting. The study by Buck (2008) also failed to find a relationship between aerobic physical fitness and orienting in a sample of preadolescent athletes.

Finally, the few studies conducted to date on the relationship between regular physical exercise and phasic alerting in children have yielded scarce and inconclusive results. Buck (2008) found no effect of increased aerobic fitness on the alerting network in preadolescent athletes. However, a study by Verburgh et al. (2014) showed that highly skilled soccer players responded faster to a warning signal than amateur soccer players. Other studies have shown a positive relationship between deliberate practice of sports with high perceptual and decision-making demands and tonic alertness or vigilance (Ballester et al., 2015, 2018, 2019).

As described in the above review, the association between regular exercise and attentional functioning in general, and executive control in particular, has generally been approached by analyzing each cognitive function in isolation. These studies have been mainly focused on samples of older adults (e.g., Colcombe and Kramer, 2003; Bixby et al., 2007; Prakash et al., 2011) and, to a lesser extent, on younger adults (e.g., Stroth et al., 2009; Kamijo and Takeda, 2010) and children (Chaddock et al., 2011a, b; Davis et al., 2011). In addition, most studies mentioned above have highlighted the mediating role of cardiovascular or aerobic fitness when explaining the beneficial effect of regular exercise on executive attention (e.g., Guiney and Machado, 2013).

The main novelty in our study resides is the exploration of the link between deliberate practice of a predominantly open-skill sport (soccer) and executive control in interaction with other attentional networks in children. Our research is based on previous findings, which generally showed some benefits of regular physical exercise on various functions linked to executive control. Accordingly, we expect to find better executive control in a group of children with deliberate soccer practice compared to a group of children with no practice.

## Materials and Methods

### Participants

We conducted a cross-sectional study on a sample of 131 male children aged 10–12 years [*M*_age_ = 10.87 years old, standard deviation (SD) = 0.85]. Seventy of them were non-athlete primary school students (*M*_age_ = 10.84 years old, SD = 0.84) who fulfilled our previously defined inclusion criteria: no systematic sport practice or less than 5 h per week of sport participation outside school. These criteria were established according to data obtained from a survey on physical activity and sport practice habits. In order to guarantee systematization of practice and homogeneity of training, sleeping, and study habits, the athlete group, 61 children (*M*_age_ = 10.90 years old, SD = 0.86), were recruited from several U10 and U12 teams enrolled in a youth elite soccer academy of La Liga club in the Valencia region of Spain.

All participants self-reported normal or corrected-to-normal vision. Statistical analyses were conducted on data from only 113 participants, after excluding those who did not perform the task appropriately (a higher than 25% error rate—4 participants) and 4 non-athletes who practiced sport for more than 5 h per week.

A sensitivity analysis conducted with G^∗^ Power (Faul et al., 2007) showed that with our sample size divided into two groups (i.e., soccer players, 59, vs non-athletes, 54), the minimum effect size that could be detected for the between-group differences regarding each attentional function for α = 0.05 and 1 – β = 0.80 was *f* = 0.188 (i.e., minimum detectable effect).

The Research Institute of Sport Sciences of the Catholic University of Valencia granted ethical approval for this study (code UCV/2015/2016/22), which also complied fully with the 1964 Declaration of Helsinki and its later amendments. Participation in the study was voluntary; all participants and their parents or legal guardians were properly informed about the risks and benefits of the study prior to any data collection and signed an institutionally approved informed consent form. The participants were also informed of their right to leave the experiment at any time.

### Procedure

All the tests were performed in a single experimental session lasting approximately 45 min in the afternoon during normal school hours or during the training hours of the soccer players. First, the group of non-athletes completed the questionnaire on their physical-sports activity practice habits, in which they had to specify whether they practiced any sports outside their school physical education classes, which sport or sports they practiced, if any, and the number of hours they performed this activity per week. This questionnaire was not completed by the group of soccer players because they all had three training sessions a week lasting approximately 90 min, in addition to a weekend match lasting approximately 90 min. Participants were given an explanation of the ANT-I (Attentional Network Test–Interactions) task developed by Callejas et al. (2004) and were then asked to complete it.

### Cognitive Task

The ANT-I is a modified version of the Attentional Network Task, known as ANT (Fan et al., 2002). It was developed to assess the independent functioning of the three attentional networks (alerting, orienting, and executive control or inhibitory control), as well as the potential interactions between them; it combines a spatial orienting task (Posner, 1980), a flanker task (Eriksen and Eriksen, 1974), and an audio signal to assess the functioning of the phasic alerting network. The efficiency of each network and its interactions are measured by registering reaction times (RTs) and response accuracy (i.e., percentage of errors). Considering the complex relationships that underlie the perceptual contexts that characterize most team sports (for a review of the benefits of using the ANT-I vs the ANT, see Ishigami and Klein, 2010), the ANT-I seems a suitable task to measure attentional functioning.

Data collection took place in specially equipped data collection rooms, under dimly lit conditions with no distracting noises. Participants were seated approximately 60 cm from a 15-inch monitor in which the attentional task stimuli were presented using E-Prime software (Schneider et al., 2002). Participants wore headphones through which they could hear the acoustic warning signal. The experiment began by displaying instructions to perform the task, while the researcher in charge of the experiment provided any necessary additional information.

**FIGURE 1:**
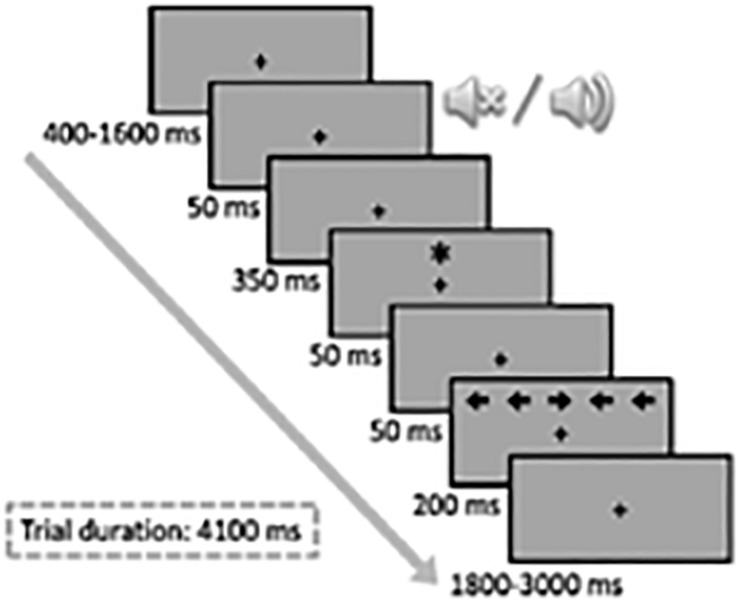
Experimental procedure and stimuli sequence in the Attentional Network Test—Interactions task.

Participants were encouraged to respond as quickly and accurately as possible by pressing the “C” (left) or “M” (right) key on the keyboard depending on the direction of the target stimulus (a central target arrow 0.55° long pointing either left or right), which was flanked by two other irrelevant arrows identical to the target on each side (0.06° away from each other). In each trial, the target arrow was preceded by an acoustic alerting tone (2,000 Hz and 50 ms) and/or a spatial orienting visual cue (an asterisk 0.6° in size and 50 ms). Participants were strongly encouraged to fixate the fixation point (variable duration of 400–1,600 ms) throughout the entire trial. The sequence of events for each trial is shown in Figure 1. The interference variable was defined according to the congruency of the direction of the flankers and target arrows: congruent trials (50% of trials), in which the target was flanked by arrows pointing in the same direction, and incongruent trials (the remaining 50% of trials), in which the flanking arrows and the target pointed in opposite directions. The orienting signal was presented above or below the fixation point in two-thirds of the trials. Three orienting conditions were thus established according to the presence of the cue: cued location trials, when the cue was presented at the same location as the target; uncued location trials, when the cue was presented at the opposite location to the target; and absence of cue, or no cue trials, when no cue was presented. The alerting signal was presented before the onset of the target in only half of the trials. The alerting variable was established according to the presence (tone) or absence (no tone) of the acoustic warning signal. The target was presented until the participants responded or for 1,700 ms. After the response, or after the allocated time had elapsed, the fixation point was presented for a variable length of time (depending on RTs and the duration of the initial display for that trial) so that all trials were equally long (4,450 ms). Initially, participants completed a practice block of two trials (including feedback about the response), followed by six experimental blocks of 48 trials each (without feedback), with resting intervals of about 1 min between them. Participants performed the ANT-I for approximately 25 min.

### Statistical Analyses

Descriptive statistics (mean ± SD) were used to collect data on various aspects of the sample and groups. Normal distribution of data and the assumptions of sphericity were examined using Shapiro–Wilk and Mauchly tests, respectively. We conducted a mixed repeated-measures MANOVA on the data of each dependent variable (RTs and accuracy), with Deliberate Sport Practice (soccer players vs non-athletes) as a between-group factor and the other independent variables (Alerting: tone, no tone; Orienting: uncued, no cue, cued; and Congruency: congruent, incongruent) as within-participant factors. A first analysis was conducted by means of repeated-measures ANOVAs on both mean RTs and error percentages, including Alerting signal (no tone/tone), Orienting visual cue (invalid/no cue/valid), and Congruency (congruent/incongruent), in order to establish whether the usual pattern of results was observed. *Post hoc* analyses (paired *t*-tests) were conducted to further explore significant interactions, with the Tukey correction for multiple comparisons. The alpha level was set at *p* < 0.05 for univariate ANOVAs and repeated-measures ANOVAs. The partial eta squared (η_*p*_^2^) effect size was also reported, indicating small (η_*p*_^2^ > 0.01), moderate (η_*p*_^2^ > 0.06), or strong (η_*p*_^2^ > 0.14) effects (Field, 2009).

## Results

### Descriptive Analysis of the Attentional Variables

Data from incorrect response trials (2.68%), or those whose RTs were 2.5 SD above (2.63% lapses) or below (0.05% anticipation) the average RTs for each participant, were not included in statistical analyses of RTs. Descriptive results are displayed in Table 1.

**TABLE 1:** Mean (M) ± SD reaction times and error percentage (in parenthesis) for each experimental condition and group.

		No tone			Tone		
		Uncued	No cue	Cued	Uncued	No cue	Cued
Non-athletes (*n* = 54)	Congruent	802 ± 183 (2.31 ± 3.90)	815 ± 184 (2.93 ± 3.73)	758 ± 158 (2.31 ± 4.06)	744 ± 131 (2.01 ± 3.82)	713 ± 128 (1.77 ± 3.36)	699 ± 157 (2.16 ± 3.65)
	Incongruent	981 ± 221 (7.95 ± 8.34)	978 ± 227 (10.34 ± 12.72)	907 ± 207 (8.41 ± 9.47)	936 ± 195 (10.34 ± 8.32)	922 ± 200 (9.65 ± 10.14)	857 ± 204 (7.95 ± 10.18)
Soccer players (*n* = 59)	Congruent	657 ± 101 (1.69 ± 2.98)	660 ± 110 (2.05 ± 4.01)	628 ± 97 (1.41 ± 2.72)	619 ± 90 (2.19 ± 4.36)	590 ± 99 (1.69 ± 3.17)	579 ± 102 (0.92 ± 2.66)
	Incongruent	780 ± 125 (7.27 ± 8.49)	766 ± 125 (5.93 ± 7.20)	720 ± 122 (4.52 ± 6.39)	761 ± 113 (9.53 ± 10.82)	716 ± 108 (7.84 ± 10.03)	671 ± 92 (6.36 ± 8.02)
							

### Analysis of Attentional Functioning

Attentional effects were evaluated by means of 2 (Alerting) × 3 (Orienting) × 2 (Congruency) repeated-measures ANOVAs of mean RTs and error percentages. The analysis of RTs showed the typical functioning patterns of each of the attentional networks, Congruency, *F*(1,112) = 486.81, *p* < 0.001, η_*p*_^2^ = 0.81; Alerting, *F*(1,112) = 145.83, *p* < 0.001, η_*p*_^2^ = 0.57; and Orienting, *F*(2,224) = 169.72, *p* < 0.001, η_*p*_^2^ = 0.60. Similarly, executive control was modulated by the other networks in RT, Alerting × Congruency, *F*(1,112) = 11.68, *p* < 0.001, η_*p*_^2^ = 0.09, and Orienting × Congruency, *F*(2,224) = 23.36, *p* < 0.001, η_*p*_^2^ = 0.17. A significant interaction was also found between Alerting and Orienting, *F*(2,224) = 11.77, *p* < 0.001, η_*p*_^2^ = 0.09, in RT. Regarding the analysis on response accuracy, the main effects of Congruency, *F*(1,112) = 101.11, *p* < 0.001, η_*p*_^2^ = 0.47, and Orienting, *F*(2,224) = 9.90, *p* < 0.001, η_*p*_^2^ = 0.08, were replicated, but not those of Alerting, *p* = 0.102. We also observed the typical interactions between Alerting × Congruency, *F*(1,112) = 9.03, *p* = 0.003, η_*p*_^2^ = 0.08, and Orienting × Congruency, *F*(2,224) = 4.33, *p* = 0.014, η_*p*_^2^ = 0.04; the Alerting × Orienting interaction, *F*(2,224) = 2.93, *p* = 0.055, η_*p*_^2^ = 0.03, was marginally significant. Therefore, the task showed the usual pattern of results.

### Analysis of the Attentional Differences Between Non-Athletes and Soccer Players

More in line with the main aims of this study, the results of the 2 (Deliberate Sport Practice) × 2 (Alerting) × 3 (Orienting) × (2 Congruency) MANOVA revealed a main effect of Deliberate Sport Practice on RTs, *F*(1,111) = 38.01, *p* < 0.001, η_*p*_^2^ = 0.25, but not on response accuracy (*p* = 0.145). Soccer players were 164 ms faster than non-athletes and also tended to show higher accuracy (4.28 and 5.67% error rates, respectively).

More importantly, our results revealed a positive association between deliberate sport practice and executive control. Soccer players showed reduced conflict compared to non-athletes with both response speed [*F*(1,111) = 28.01, *p* < 0.001, η_*p*_^2^ = 0.20] and response accuracy, although in this case, the difference was not significant (*p* = 0.181).

**FIGURE 2:**
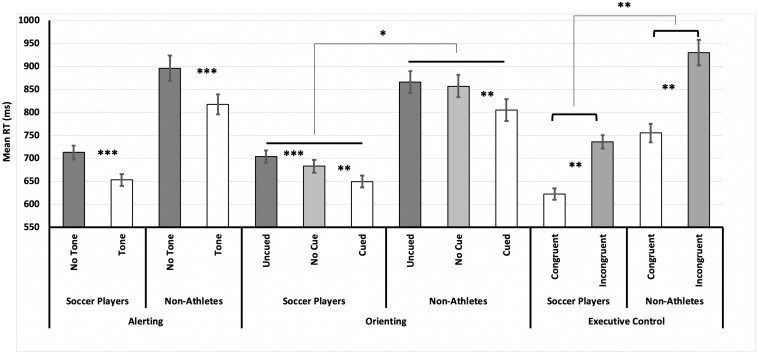
Mean reaction time (RT) for each experimental condition used to measure each attentional function, as a function of Deliberate Sport Practice. For alerting, only no-cue trials were used to compute the means. Note that soccer players were faster in general than non-athletes and showed reduced interference (i.e., reduced difference between congruent and incongruent conditions). Error bars represent the standard error of the mean. *Indicates significant differences between groups and attentional conditions (*p < 0.05, **p < 0.001, ***p < 0.0001).

We also observed between-group differences in the functioning of attentional orienting, both in response speed, *F*(2,222) = 4.31, *p* = 0.015, η_*p*_^2^ = 0.04, and in response accuracy, *F*(2,222) = 3.85, *p* = 0.023, η_*p*_^2^ = 0.03. However, as shown in Figure 2, *post hoc* analyses showed no differences between soccer players and non-athletes in the overall orienting effect (uncued–cued), *p* = 0.320. Between-group differences (with faster RTs in the group of soccer players) were particularly evident in the no-cue condition, in which no temporal or spatial signal was presented that could be used as a reference for preparation. A similar effect was found in response accuracy, with a lower percentage of errors especially in the cued and no-cue conditions.

Finally, alerting was not significantly affected by Deliberate Sport Practice in either response speed (*p* = 0.070) or response accuracy (*p* = 0.072).

Finally, no relationship was observed between Deliberate Sport Practice and the interaction between executive control and the other attentional networks (all *p*s > 0.05).

## Discussion

The purpose of this study was to test the association between deliberate sports practice and attentional functioning, especially at early ages (10–12 years), given that in recent decades, there has been a growing interest in clarifying the relationship between physical exercise and cognition (McMorris, 2016). Specifically, our approach is novel in the analysis of the influence of predominantly open-skill sports (i.e., soccer) on executive control, in a context of complex interaction with other attentional networks (i.e., alerting and orienting). The study of these relationships from childhood to preadolescence is especially interesting, as it is a crucial period for sports initiation (e.g., Raya et al., 1993, in the case of soccer) and coincides with a key evolutionary stage in cognitive development in general and concerning attentional networks in particular (Casey et al., 2000; Rueda et al., 2004).

Regarding the functioning of attentional networks, our results replicated those of previous studies conducted using the ANT task (Mezzacappa, 2004) or the ANT-I (Callejas et al., 2004; Rueda et al., 2004) revealing the typical main effects of executive control, alerting, and orienting, as well as the interactions between them (Alerting × Conflict, Orienting × Conflict, and Alerting × Orienting). Our findings confirm that the ANT-I is a useful and valid tool for assessing attentional functioning in children aged between 10 and 12 years. The ANT-I is a task which demands a complex space–time interaction of visual and sound stimuli. In our opinion, it is more similar to the demands of many situations in the real environment and, specifically, in many sports setting. The complexity of the stimuli presented by the ANT-I requires the simultaneous functioning of all three attentional networks. We therefore consider that the methodological paradigm used to study the relationship between sports practice and executive control is more ecological than those previously proposed in other studies using tasks that evaluate the functioning of this network in isolation (e.g., Castelli et al., 2011; Davis et al., 2011; Chaddock et al., 2012).

Regarding the specific objectives of our study, we found that deliberate practice of an open-skill sport such as soccer was associated to faster and more accurate responses, although the accuracy result did not reach statistical significance. These findings are consistent with those reported by previous studies showing the benefits of aerobic fitness, inherent to regular sports practice, on response speed (see the meta-analysis by Voss et al., 2010). Our results are also consistent with the findings of Ballester et al. (2015, 2018) showing that deliberate soccer practice during preadolescence was positively associated with response speed, albeit those studies used a different cognitive task (i.e., the Psychomotor Vigilance Task).

More importantly, and focusing on the association between deliberate sport practice and the functioning of executive control, our results showed a lower level of conflict in the group of soccer players compared to their non-athletes counterparts. As stated in the section “Introduction,” numerous studies have explored this relationship using different paradigms. In general, a pattern of results similar to that obtained in the present study has been observed. However, most studies used tasks suitable to measure executive control in isolation, independently from other cognitive functions (see reviews by Best, 2010; Guiney and Machado, 2013; de Greeff et al., 2018; Sanders et al., 2019). We consider that between-groups differences in executive control could be explained by the two dominant hypotheses in the study of the relationship between sports practice and cognitive functioning.

It is logical to argue that, though this variable was not measured in our study, the physiological demands of regular sport practice may have led to a better aero-anaerobic physical fitness in soccer players vs non-athletes (Ballester et al., 2018). In this regard, the cardiovascular fitness hypothesis (North et al., 1990; Voss et al., 2011) postulates that higher cardiovascular capacity, which is inherent to the regular practice of physical exercise, accounts for the cognitive improvement of individuals who exercise regularly due to physiological adaptations. Some examples of this are increased VO_2_, increased brain-derived neurotrophic factor (BDNF), and increased cerebral blood flow (North et al., 1990; Voss, 2016). In fact, the reviewed literature offers ample evidence of the positive impact of an improvement in aerobic fitness on the executive function in children (Buck et al., 2008; Hillman et al., 2009; Chaddock et al., 2010, 2012; Castelli et al., 2011; Davis et al., 2011; Pontifex et al., 2011; Voss et al., 2011). However, there is also some evidence that cardiovascular fitness is not a physiological mediator for improved cognitive performance in children (e.g., see Etnier et al., 2006; Ballester et al., 2018; Sanabria et al., 2019). In our opinion, these discrepancies may be partially explained by the methodological variations between the different studies, which make their results difficult to compare.

In view of this, the observed better executive control in soccer players than in non-athletes could alternatively be explained by the cognitive component skills approach (Mann et al., 2007; Voss et al., 2010). In our case, it is important to note that optimal performance in externally paced sports such as soccer requires not only a good level of physical fitness but also the ability to quickly adapt and respond to the demands of complex and constantly changing situations. Thus, the systematic and structured practice of externally paced sports, such as soccer, involves the learning and practice of basic cognitive abilities to manage these situations. The cognitive component skills approach would imply that this learning would be transferred to other general or specific domains, as proven by previous studies (Best, 2010; Williams et al., 2011; Ballester et al., 2019). In this vein, Wang et al. (2013) observed that university students who practiced an externally paced sport (i.e., tennis) exhibited greater inhibitory control than those who practiced a self-paced sport (i.e., swimming). The specific demands of the soccer environment may require this “cognitive specialization” in attentional performance, given that players are exposed to situations where they have to select relevant stimuli in a complex environment and decide between several possible options under high time pressure (Abernethy et al., 1993; Williams et al., 1999; Voss et al., 2010).

Our results seem to show that the association between deliberate soccer practice and executive control occurs at early ages and in a complex cognitive evaluation context, where the response is conditioned by stimuli that require the simultaneous participation of other attentional networks (i.e., orienting and alerting). Likewise, we observed a connection between sport practice and the functioning of orienting. However, in-depth analyses of this interaction showed that the greatest benefit observed in the group of non-athletes was due to the fact that they were significantly slower than soccer players in the no-cue conditions, not that they processed the orienting signal differently. This is consistent with the existing literature, given that there is very little evidence linking systematic sport practice to spatial orienting. Previous studies have, rather, focused on the analysis of the acute effect of exercise on exogenous (e.g., Huertas et al., 2011, 2019; Sanabria et al., 2011; Llorens et al., 2015) or endogenous spatial orienting (e.g., [citeskum]BR53,BR54,BR55,BR56,BR57,BR58[citeekum]Pesce et al., 2002, 2003, 2004, 2007a, 2007b, 2011).

Finally, our study did not find any link between deliberate sport practice and the functioning of the alerting network. Although the existing literature is scarce and controversial, our results are in line with those described by Buck (2008) showing no between-groups differences in the alerting network (evaluated with the ANT) in preadolescent children classified according to their level of aerobic fitness. By contrast, Verburgh et al. (2014) found that, in a sample of young soccer players aged 8–16 years from teams of different levels, presenting the warning signal benefited the players in higher-level teams. Regarding vigilance, the studies by Sanabria et al. (2019) and Ballester et al. (2018) did not find that the differences in vigilance functioning were explained by the cardiovascular fitness hypothesis but, rather, that these differences were due to the type of sport practiced. This leads us to confirm again that, when analyzing the relationships between systematic sport practice and any cognitive function, it is essential to control the influence of the variables that can mediate in this relationship: both those related to the development of the physical fitness of the participants and those related to the level of expertise, skill, or experience in the practice of the sport. It would be interesting to conduct further studies analyzing phasic alerting while controlling for aerobic capacity, in order to verify whether this factor is a positive mediator of this attentional function.

Some of the controversial findings described in several studies regarding the relationship between regular sports practice and cognitive functioning could be explained by the different methodological approaches used. First, it is important to analyze the effect of the age of the participants given that, as shown by many studies, it modulates the functioning of the different attentional functions and may explain these differences. It should be noted that the orienting network (e.g., Wainwright and Bryson, 2002) and the executive control network (e.g., Rueda et al., 2004; Simonds et al., 2007) seem to become consolidated before the age of 10, whereas the alerting network seems to continue its development after this age (e.g., Rueda et al., 2004; Pozuelos et al., 2014). In view of this, it would be interesting to conduct further longitudinal studies or studies including different age groups to determine how systematic sports practice modulates the development of each network at different stages. Moreover, when explaining the association between deliberate sport practice and executive control and the rest of the attentional functions, it is important to describe the type of task used for its assessment, the level of complexity and cognitive demand, the interaction with other networks, and other aspects. In this regard, difficulties could arise when comparing our results with those obtained by Verburgh et al. (2014), given that they used the ANT, which measures alerting through visual signals, as opposed to acoustic signals used in the ANT-I and thus in our research. These differences in the specificity of the stimuli used to assess the various cognitive functions, in accordance with the cognitive component skills approach, may explain the controversy in the results of various studies (e.g., in soccer, visual warning signals are likely to be much more relevant and specific than acoustic warning signals).

Finally, we suggest that further research about the relationship between regular sports practice and cognition should consider both the role of the type of sport practiced (i.e., self-paced vs externally paced), linked to the cognitive component skills approach (Mann et al., 2007; Voss et al., 2010), and the practitioner’s physical fitness, as proposed by the cardiovascular fitness hypothesis (North et al., 1990; Voss et al., 2011). This will allow assessment of the influence of each of these potential mediating factors in the development of every cognitive function studied. In this sense, one limitation of the present study is that its methodological design does not allow us to adopt a defined position in favor of either of the two hypotheses described above. The reason is that we consider that, as shown by previous studies, the soccer player group probably had a greater cardiovascular fitness level (e.g., Vänttinen et al., 2011; Plaza-Carmona et al., 2013; Ballester et al., 2015) and a better level of development of the attentional functions (Daus et al., 1989; Abernethy et al., 2001; Farrow and Abernethy, 2003) than the non-athlete group. In short, we believe that the contextual demands to which these players are regularly subjected in training and competition may lead to physical, physiological, perceptual, and cognitive adaptations and induce changes in the functioning of executive control and other cognitive functions.

Furthermore, our results could be partly explained by the existence of a self-selection bias in the sport context, the so-called “neuroselection effect,” according to which individuals with better cognitive functioning choose more active and healthier lifestyles (e.g., Kanazawa, 2013; Belsky et al., 2015). According to this hypothesis, children who play soccer in high-level clubs could have higher development of certain cognitive abilities, such as executive control, which would facilitate their involvement in these types of open-skills sports. An important direction for further research might be to investigate the functioning of the attentional networks in different sport disciplines (self-paced vs externally paced) controlling both the athlete’s level of expertise and the athlete’s levels of cardiovascular fitness, in order to clarify their relative contribution to attentional performance. Given the cross-sectional nature of the present study, future research should also use experimental designs with random assignment of participants to the experimental and control groups in order to add more solid evidence about the causal effect of deliberate sport practice on executive functions.

## Conclusion

Our results shed new light on the advantageous connection between the deliberate practice of externally paced sports during preadolescence and both response speed and executive control, as measured in a context of interaction with other attentional networks.

However, given the cross-sectional design of our study, our findings do not allow us to confirm the underlying reasons for the advantage observed in the cognitive performance of soccer players. Different hypotheses could explain our results. First, the potential higher physical fitness level of soccer players may have an influence on neurophysiological aspects that benefit executive control, in line with the cardiovascular fitness hypothesis. Additionally, athletes’ executive control may also have been improved by repeated exposure to situations where these functions are in high demand, as proposed by the cognitive component skills approach. Finally, and according to the “neuroselection effect,” sport practitioners probably had, before starting the training program, more active lifestyles and better cognitive function than their non-athlete counterparts, which would also contribute to their better cognitive functioning.

Independently of the moderators and mediators underlying this association, these findings are particularly relevant, as they highlight the need to promote physical-sports activity at an institutional level from an early age, given its positive impact on both physical and cognitive development. They also reinforce the recommendations issued by the World Health Organization (World Health Organization [WHO], 2010) on the regular practice of physical exercise. In view of our findings, we propose that, within the recommended parameters of physical activity intensity (i.e., moderate and intense for at least 60 min per day), it would be interesting to specify the following: the activity should involve perceptual–cognitive and decision-making processes (e.g., externally paced sports such as team, combat, or racket sports). In fact, as shown by the current literature (e.g., Tomporowski and Pesce, 2019), physical-sport activities with higher perceptual–cognitive demands seem to be the most beneficial at the cognitive level.

## Data Availability Statement

The raw data supporting the conclusions of this article will be made readily available to all qualified researchers. Requests for access to the data should be addressed to CM at consuelo.moratal@ucv.es.

## Ethics Statement

The studies involving human participants were reviewed and approved by Research Ethical Committee from Catholic University of Valencia (UCV/2015/2016/22). Written informed consent to participate in this study was provided by the participants’ legal guardian/next of kin.

## Author Contributions

CM: conception and design of the study, the data acquisition, analysis, and interpretation, drafting of the article and critical revision for important intellectual content with specific contribution regarding the physical activity–attention relationship, final approval of the version to be published, and agreement to be accountable for all aspects of the research. JL: contribution to the conception of the study, creation of the attentional test, the data analysis and interpretation, drafting and critical revision of the article for important intellectual content with specific contribution regarding attentional functioning issues, final approval of the version to be published, and agreement to be accountable for all aspects of the research. RB: data interpretation, drafting and critical revision of the study for important intellectual content with specific contribution regarding systematic sport practice issues, contribution to drafting the article, final approval of the version to be published, and agreement to be accountable for all aspects of the research. FH: main role in the conception and design of the study with a relevant role in project coordination and data acquisition, interpretation and critical revision of the article with specific contribution regarding the physical activity–attention relationship, final approval of the version to be published, and agreement to be accountable for all aspects of the research.

## Conflict of Interest

The authors declare that the research was conducted in the absence of any commercial or financial relationships that could be construed as a potential conflict of interest.
